# Recent progress in electrospun nanomaterials for wearables

**DOI:** 10.1063/5.0088136

**Published:** 2022-06-28

**Authors:** Riddha Das, Wenxin Zeng, Cihan Asci, Ruben Del-Rio-Ruiz, Sameer Sonkusale

**Affiliations:** Department of Electrical and Computer Engineering, Tufts University, 200 Boston Avenue, Medford, Massachusetts 02155, USA

## Abstract

Wearables have garnered significant attention in recent years not only as consumer electronics for entertainment, communications, and commerce but also for real-time continuous health monitoring. This has been spurred by advances in flexible sensors, transistors, energy storage, and harvesting devices to replace the traditional, bulky, and rigid electronic devices. However, engineering smart wearables that can seamlessly integrate with the human body is a daunting task. Some of the key material attributes that are challenging to meet are skin conformability, breathability, and biocompatibility while providing tunability of its mechanical, electrical, and chemical properties. Electrospinning has emerged as a versatile platform that can potentially address these challenges by fabricating nanofibers with tunable properties from a polymer base. In this article, we review advances in wearable electronic devices and systems that are developed using electrospinning. We cover various applications in multiple fields including healthcare, biomedicine, and energy. We review the ability to tune the electrical, physiochemical, and mechanical properties of the nanofibers underlying these applications and illustrate strategies that enable integration of these nanofibers with human skin.

## I. INTRODUCTION

Wearables encompass miniaturized electronic devices worn directly on human skin for sensing a diverse range of biophysical and biochemical signals^1–6^ or to provide convenient human-machine interfaces (e.g., smart watches). They facilitate continuous health monitoring^7–10^ and can even serve as a way to generate or store energy^11–14^ all the while eliminating the need for traditional, bulky, and rigid electronics.^15^ However, engineering wearables that can seamlessly integrate with the human body is a daunting task.^16,17^ Some of the key attributes particularly required of the materials for wearable devices are skin conformability,^12,18,19^ breathability,^20–22^ and biocompatibility.^23–25^ Also critical is the ability to tune the mechanical, electrical, and chemical properties of the material. Electrospinning provides a versatile platform to potentially address these challenges by producing nanofibers from a polymer solution using an electric field^26–33^ and assemble them in various formats such as films, threads, or mats. The physiochemical characteristics of the nanofibers can be engineered by varying the inherent core material of the polymer and the solvent, incorporation of additives, and tuning the process parameters during electrospinning.^34,35^

**FIG. 1.**
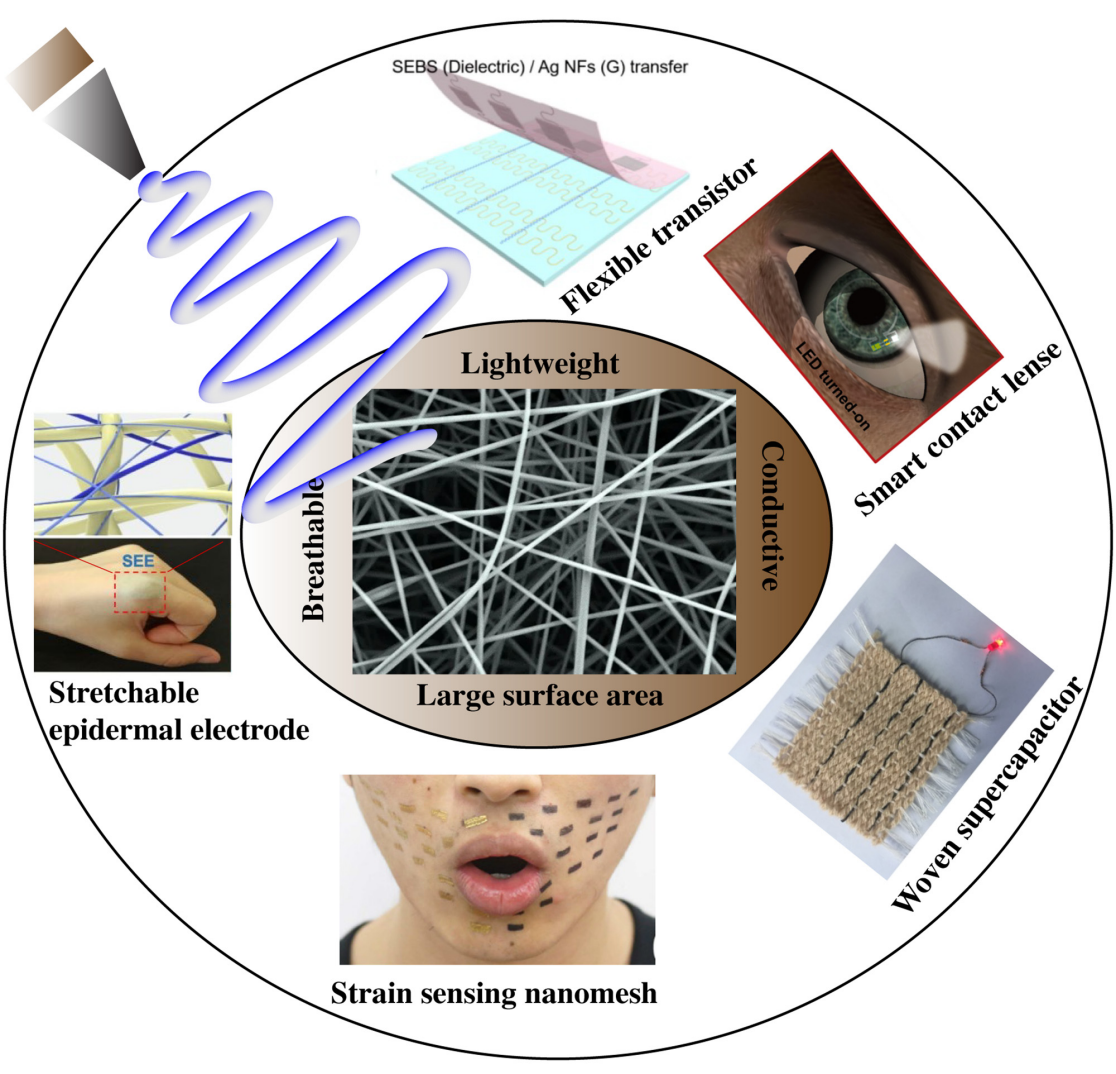
Electrospun nanofiber based wearable devices: a stretchable epidermal electrode, a strain sensing nanomesh, a woven supercapacitor, a smart contact lens, and a flexible transistor. Reproduced with permission from Fan *et al.*, Nanoscale **12**, 16053 (2020). Copyright 2020 Royal Society of Chemistry. Reproduced with permission from Wang *et al.*, Sci. Adv. **6**, ▪ (2020). Copyright 2020 Authors, licensed under a Creative Commons Attribution (CC BY) license. Reproduced with permission from Park *et al.*, Sci. Adv. **4**, eaap9841 (2018). Copyright 2018 Authors, licensed under a Creative Commons Attribution (CC BY) license. Reproduced with permission from Sun *et al.*, Materials **12**, 273 (2019). Copyright 2019 Authors, licensed under a Creative Commons Attribution (CC BY) license, Reproduced with permission from Kim *et al.*, Nano Lett. **21**, 5819 (2021). Copyright 2021 American Chemical Society.^74,82,124,161,183^

The concept of electrospinning can be traced back to the study performed by Gilbert in 1600,^36^ when he observed the formation of a cone shaped water droplet in the presence of an electric field. In 1902, Cooley^37,38^ and Morton^39^ filed multiple patents on the setup for performing electrospinning. However, first ever known implementation of electrospun nanofibers occurred in the Soviet Union in 1938 for the development of air filters, known as “Petryanov filters,” for capturing aerosol particles. Remarkable studies were performed by Geoffrey Ingram Taylor to mathematically predict the cone shape of the polymer fluid under an electric field during electrospinning and were published in multiple reports between 1964 and 1969.^40–42^ Despite tremendous progress, the early 1990s truly marked the beginning of electrospinning, when the term “electrospinning” was popularized by several research groups to describe the fabrication process of long, continuous nanofibers from the polymer melt or solution.^41,43,44^ Since polymers are used as the base material for electrospinning, one has access to several material choices, which can be incorporated individually or in combination to achieve nanofibers with distinct properties such as biocompatibility,^45–49^ flexibility,^50–52^ conductivity,^53–56^ breathability,^20,21,57,58^ and porosity.^59–61^ In this review, we have mainly focused on wearable devices that are made using electrospinning (Fig. 1) and further highlighted their applications in multiple fields, including healthcare, biomedicine, and energy.

## II. FUNDAMENTALS OF ELECTROSPINNING

**FIG. 2.**
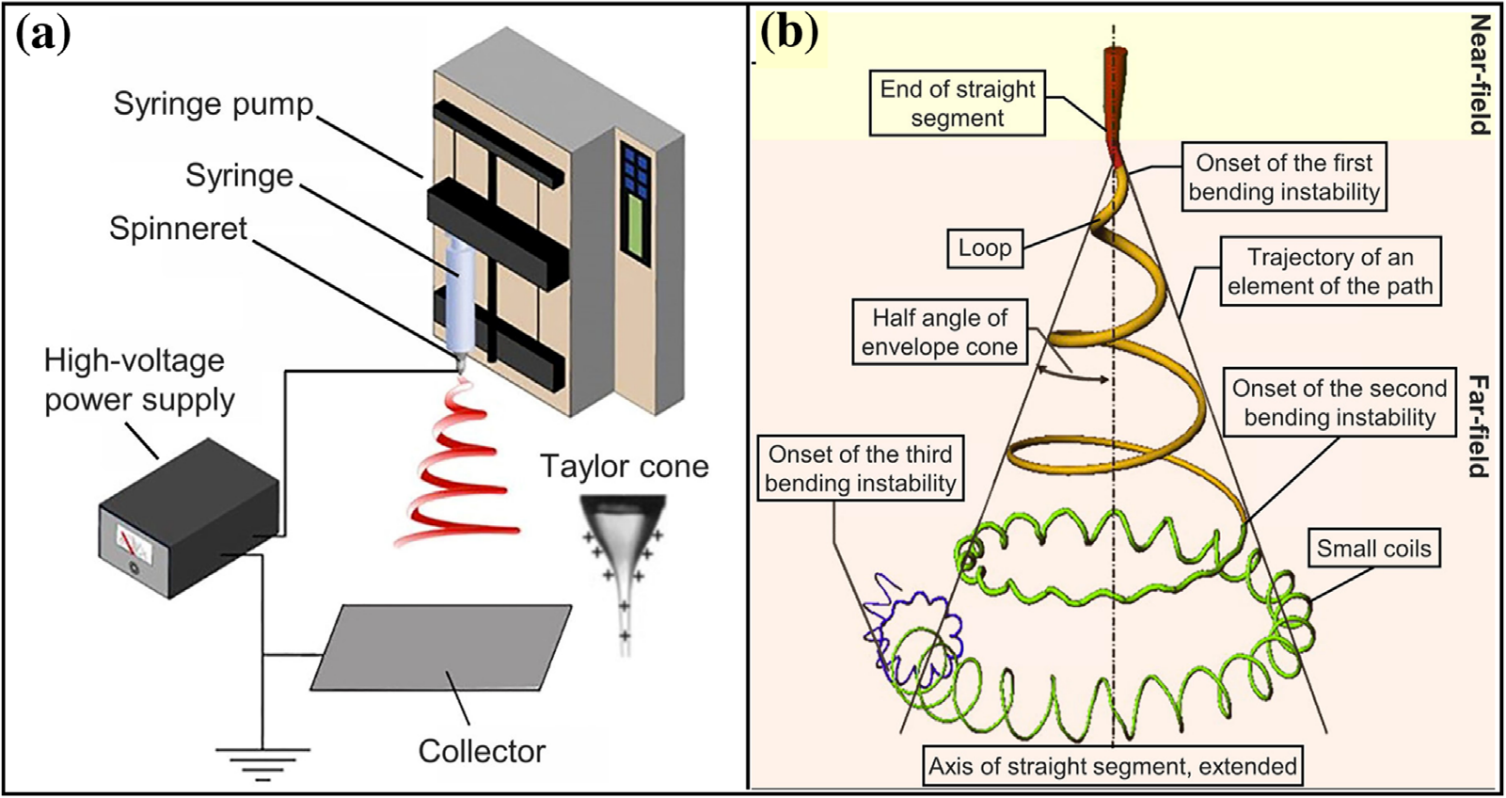
Schematic diagram of (a) a basic setup of electrospinning and (b) the path of an electrospun jet with bending instability. Reproduced with permission from Xue *et al.*, Chem. Rev. **119**, 5298 (2019). Copyright 2019 American Chemical Society.^27^

Electrospinning involves drawing a liquid polymer jet in the presence of an electric field that leads to stretching and thinning of the jet to a few hundred nanometers diameter followed by solidification and deposition on a grounded electrode. It is important to understand the fundamental principles to enable better control of the processing parameters, which in-turn allow generation of materials with desirable chemical and mechanical properties for different wearable applications. This section summarizes the basic setup and principles of each stage of electrospinning [Fig. 2(a)]. First, a polymer solution (molten polymer chips and/or dissolved in a suitable solvent) is fed through a syringe pump at a constant and adjustable rate to a spinneret, which is usually a hypodermic needle with a blunt tip. Initially, the droplet forms a pendant shape due to surface tension, followed by a separation between positive and negative charges in the liquid due to its potential difference created between the spinneret and the fiber collector. The charges in the same sign as the spinneret tend to accumulate at the pendant surface due to electrostatic repulsion and as a result, the pendant shape is deformed into a cone shape called as a Taylor cone.^40,41,62,63^ The critical voltage required for formation of the cone shape depends on the properties of the polymeric solution. For instance, a higher critical voltage is required to overcome the high surface tension in solutions with high viscosity. Subsequently, the jet is ejected from the apex of the cone and accelerated in the direction of the electric field. Various mathematical models based on the electrohydrodynamic theory have been reported to describe and predict the charged jet behavior to achieve a deep understanding of the mechanism.^29,31,64,65^ Briefly, the electrostatic interaction between the charges generates an upward and downward force leading to a net lateral electrostatic force perpendicular to the jet axis, causing a whooping instability and bending of the jet in the form of a spiral shape. The jet gets elongated at the loops because of the bending instability as shown in Fig. 2(b) and decreases in the diameter to the nanometer range. Higher voltage of the electric field causes rapid growth of the instability, increased bending and stretchability, and finer nanofiber formation. Solidification of the jet is caused either by cooling of the polymer jet (in the case of melt electrospinning) or rapid drying of the solvent (in the case of regular electrospinning). This emphasizes upon the importance of the solvent, and a faster evaporating solvent will ease the solidification process significantly as compared to their nonvolatile counterparts. The final step involves collection of the solidified fibers onto a grounded electrode. A slight variation of electrospinning also has been practiced by using a low viscous polymeric solution in the same setup to spray into the form of particles instead of formation of long continuous fibers. This process is called electrospraying. In many instances, electrospinning and electrospraying are combined to fabricate surface treated nanofibers.^18,66^

## III. WEARABLE SENSORS OF BIOPHYSICAL SIGNALS

Nanofibers fabricated by electrospinning are promising materials for on-skin biosensing applications owing to their high porosity and high surface/volume ratio. The porosity of nanofibers exhibits increased breathability and permeability to gas and water vapor, making them ideal substrates for the skin interface and to functionalize them to serve as sensors for different biophysical signals. This section summarizes different materials for electrospun nanofibers for personalized health monitoring and the fundamental properties of these nanofibers that enable these functions.

### A. Electrophysiological signal sensing

Monitoring biopotentials, such as electrocardiogram (ECG) and electromyogram (EMG), is central to many wearable applications. For example, continuous ECG monitoring can be used to monitor abnormal heart rhythms related to arrythmia or to detect signs of heart failure. Similarly EMG can be used as means to perform human machine interface especially in the case of prosthetics. Multiple research strategies based on conductive electrodes have been developed to monitor and record biopotential signals with high signal to noise ratio and reduced motion related artifact. One strategy is using conductive gels as skin-electrode interfaces. While these are able to detect and record these signals reliably, they are quite susceptible to drying over time making them less reliable for monitoring. Moreover, the use of gel increases the chance of an allergic reaction and even increases discomfort to the user for long-term chronic applications. Thinning the electrodes so as to make them flexible potentially provides a pathway to perform recording without the need for a gel electrolyte for reason explained below. This is revolutionary as it can allow continuous monitoring. This is possible because thin flexible electrodes adhere intimately with the skin even under motion. However, the electrodes are still fully conformal as they miss out on the various ceases, folds, and ridges of the skin and without the use of conductive gel make it very sensitive to motion artifacts and accidentally peeling off from the skin. Moreover, they are made from continuous traces of metal such as gold or carbon, making the skin underneath less breathable and, hence, ill-suited for long term monitoring.

By virtue of ultra-low thickness, shape conformability, and breathability, electrospun nanofibers are more promising as on-skin electrodes for biopotential monitoring. Since these nanomeshes are made from nanofibers, which are nanometer thick and are extremely flexible, they form truly intimate interfaces with skin covering all wrinkles. Porosity of the nanofibers improves surface contact and increase adhesion. Moreover, it makes the nanomesh electrodes breathable, a key attribute for any devices placed on the skin for long term.

**FIG. 3.**
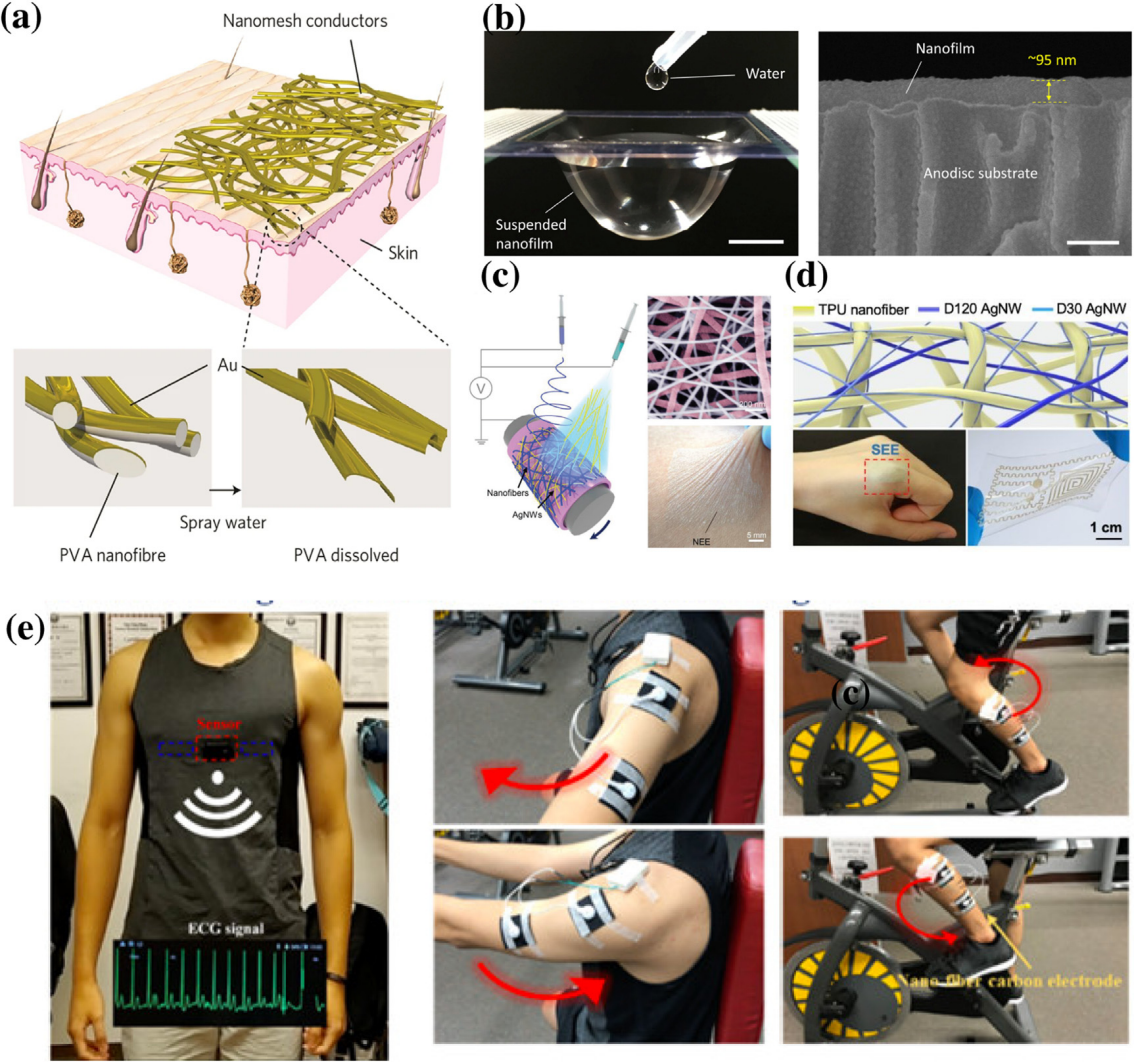
(a) Schematic diagram of on-skin conductive nanomesh fabricated by Au evaporation onto electrospun PVA nanofibers, (b) photograph of a suspended PU-PDMS nanofilm holding a liquid (scale bar, 10 mm) and cross-sectional SEM image of the nanofilm coated with 70-nm-thick Au to achieve conductivity (scale bar, 200 nm), (c) fabrication process and SEM image of NEE with a picture of the NEE attached on the forearm to illustrate the conformal contact, (d) photograph adhering on finger and twisted circuit on PDMS and the microsopic image of the SEE, and (e) schematic diagram of the application of the nanofiber-carbon electrode in measuring the ECG and EMG signals. Reproduced with permission from Wang *et al.*, NPG Asia Mater. **13**, 22 (2021). Copyright 2021 Authors, licensed under a Creative Commons Attribution (CC BY) license. Reproduced with permission from Wang *et al.*, Proc. Natl. Acad. Sci. U. S. A. **118**, e2111904118 (2021). Copyright 2021 Authors, licensed under a Creative Commons Attribution (CC BY) license. Reproduced with permission from Liu *et al.*, Small **15**, 1900755 (2019). Copyright 2019 Wiley-VCH GmbH. Reproduced with permission from Fan *et al.*, Nanoscale **12**, 16053 (2020). Copyright 2020 Royal Society of Chemistry. Reproduced with permission from C.-Y. Huang and C.-W. Chiu, ACS Appl. Electron. Mater. **3**, 676 (2021). Copyright 2021 American Chemical Society.^18,70,74,76,98^

These ultra-thin films, popularly known as nanomesh conductors, can be electrically interfaced with human skin via epidermal electrodes to transduce the biopotential signals.^67–70^ These nanomesh sensors are composed of a flexible and breathable substrate used to attach it to skin and covered with a conductive coating on the top. Mostly, gold (Au) is used as the conductive coating due to its high conductivity and biocompatibility. In one example, the electrospun nanomesh made of water dissolving polymer poly(vinyl alcohol) (PVA) coated with a thin Au layer (70–100 nm thick) was tattooed on human skin, followed by dissolution of the underneath layer of PVA in water [Fig. 3(a)] leaving behind just gold nanomesh. These conductive nanomesh recorded stable ECG and EMG signals comparable to commercial silver/silver chloride (Ag/AgCl) gel electrodes. Moreover, the nanomesh did not cause any inflammation and exhibited high breathability in dermatitis evaluation, when compared to the conventional skin patches of silicone and parylene.^69^ In another study, a self-adhesive free-standing film of PDMS and electrospun PU nanofibers coated with Au was fabricated for long term biopotential monitoring [Fig. 3(b)].^70^ This film allowed one to record ECG signals with a high signal to noise ratio of 34 db for a long time of 1 week owing to its ultra-thinness (∼95 nm) and strong adhesive property (159 *μ*J/cm^2^).

Although gold electrodes are highly efficient, they are costly, cannot be reused, and are conspicuous. As an alternative, a washable and non-disposable transparent electrode can be fabricated by using graphene as a conductive material.^67,71,72^ In one report, annealed phenolic resin (PR) was electrospun onto graphene grown by chemical vapor deposition (CVD) that was subsequently semi-embedded into a styrene–ethylene–butylene–styrene (SEBS) elastomer to exhibit excellent morphological and mechanical integrity similar to a bird's nest.^73^ Another approach to fabricate the nanomesh conductor involves using a single layer nanonetwork epidermal electrode (NEE) that can be fabricated by simultaneous electrospinning and electrospraying, instead of using a separate conductive layer [Fig. 3(c)]. This technique was originally used for fabrication of the nanofilm blended with nanoparticles. Using this technique, a homogeneous convoluted network of silver nanowires (AgNWs) was added into electrospun polyacrylonitrile (PAN) nanofibers for stable ECG monitoring by conveniently attaching the nanomesh to human skin.^18^ This NEE was 125 nm thick and highly conductive with a low sheet resistance of ≈ 4 Ω sq^−1^. Another work utilized the same mechanism to fabricate a stretchable epidermal electrode (SEE) by using thermoplastic urethane (TPU) instead of PAN [Fig. 3(d)].^74^

Carbon electrodes offer a cheaper alternative to noble metals as electrodes for long term biopotential monitoring.^71,72,75^ However, carbon electrodes may exhibit high contact impedance with the skin. One way to reduce the contact impedance is to apply Ag/AgCl gel between the electrode and the skin. However, conductive gels are not suitable for long term biopotential monitoring due to rapid drying as explained earlier. A better solution was provided by the depositing electrospun fiber layer onto the carbon electrode to fabricate a composite layer. The fiber depositor surface was coated with nanodispersed carbon black (CB) and reduced graphene oxide (rGO). A blend of PVDF/PEDOT/PSS was electrospun on the coated surface to fabricate a nanofiber carbon electrode. The nanofiber membrane layer improves the skin contact area and hydrophobicity. Higher the hydrophobicity, the greater is the contact angle with water making the electrode strongly resistant to water and stain contamination.^76^ The skin-electrode impedance of the carbon electrode without the nanofiber membrane after 20 washing cycles was found to be higher than that of the nanofiber carbon electrode due to dust accumulation and reduction of viscosity. This electrode then was integrated with smart body suit for measuring ECG and EMG as shown in Fig. 3(e).

### B. Wearable motion sensors

Another class of wearables featuring stretchable conductive peizoresistors is extensively utilized in motion, strain, and pressure sensing. In this section, we have reported some recent works on highly stretchable and sensitive wearable sensors fabricated by electrospinning that can detect physical activities (for large strains ε > 100%) such as stretching and bending, and those that can also detect delicate movements such as heartbeat and facile micro-expressions (smaller strain ε ≈ 0.1%). Conventionally, metal and semiconductor films have been used for strain sensing; however, their utility for highly sensitive kinematic sensors are limited due to their brittle and rigid physical properties that reduce skin conformability.^77^ Nanofibers are excellent alternatives satisfying the requirements of on-skin strain gauge with required porosity/permeability and thickness less than 2 *μ*m. They are capable to sustain large deformations (>50% strain) during complex human activities. Typically, these nanomesh strain gauges are bilayer substrates composed of a stretchable nanofiber layer and a conductive layer. Strain is measured by the fractional change in the electrical resistance of the conductive layer with elongation. Sensitivity of these strain gauges can be carefully programmed by modulating electrical junctions between the conductive and stretchable layers. Some of the major strategies to fabricate a nanomesh strain gauge are described in this section.

**FIG. 4.**
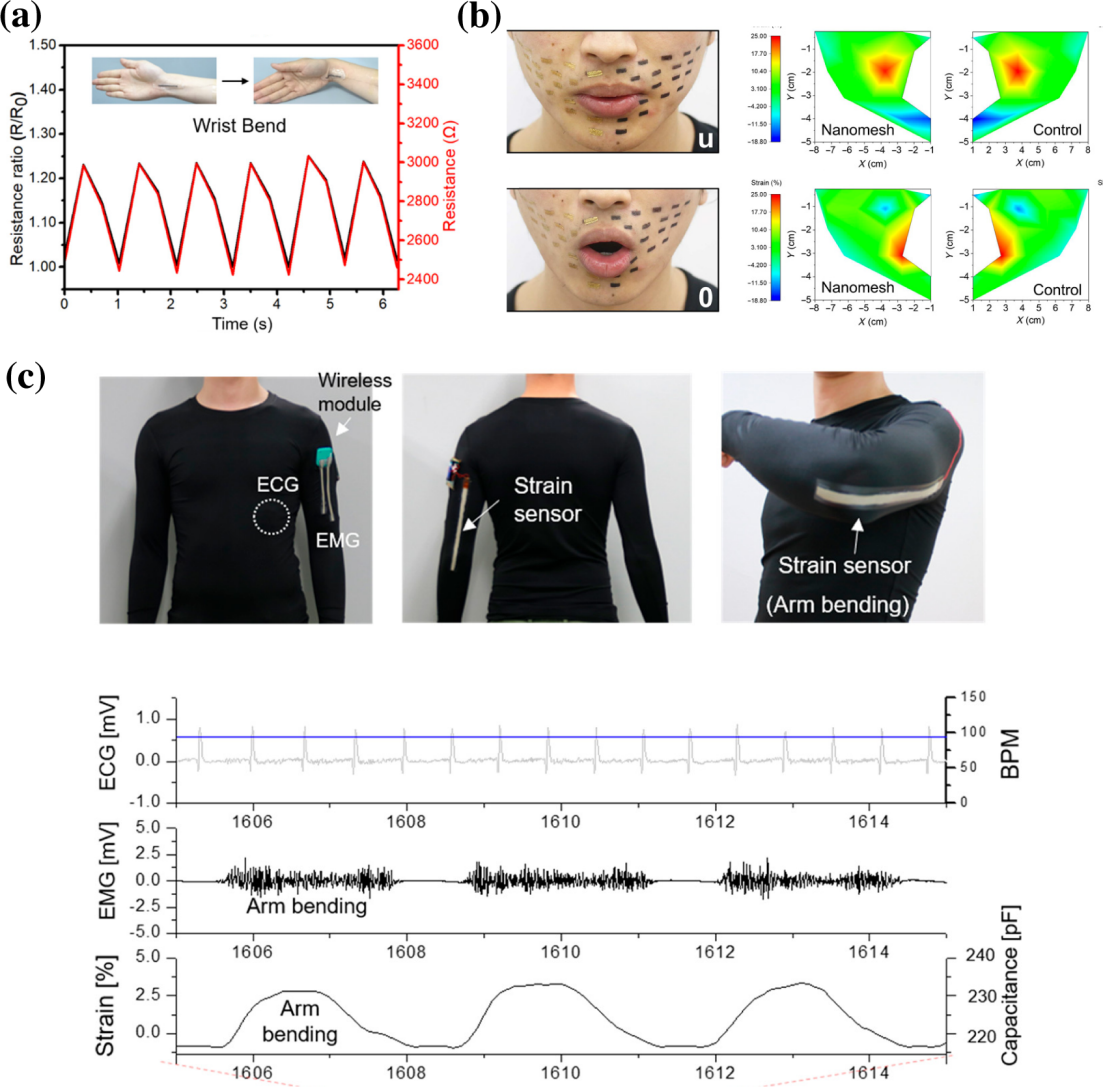
(a) Applications of the CNTs/PU helical yarn as a strain sensor to monitor the resistance changes during wrist bending. (b) Photograph of face and facile strain mapping during speech of “u” and “o.” (c) Stretchable multimodal sensing suit with the wireless transmission module for EMG, motion, and ECG signals in the real-time monitoring while lifting the 3 kg weight and subsequent release. Reproduced with permission from Gao *et al.*, ACS Nano **14**, 3442 (2020). Copyright 2020 Americal Chemical Society. Reproduced with permission from Wang *et al.*, Sci. Adv. **6**, ▪ (2020). Copyright 2020 Authors, licensed under a Creative Commons Attribution (CC BY) license. Reproduced with permission from Jin *et al.*, ACS Nano **13**, 7905 (2019). Copyright 2019 Americal Chemical Society.^80,82,85^

Polyurethane (PU) represents an important class of material due to its lightweight, high flexibility, and easy integration into functional devices or textiles.^78,79^ PU can be combined with a conductive layer, such as carbon nanotubes (CNTs), either by a simple twisting mechanism or electrospinning to fabricate composite helical yarn with electrical conductivity and ultrastretchable properties. A simple adjustment in the amount of CNT facilitates rapid response strain sensing as shown in Fig. 4(a).^80^ This multimodal platform was attached on human skin to spatially map the pressure and strain distribution during regular movements such as finger, wrist bending, and cheek bulging. Additionally, this platform was also utilized for speech recognition and swallowing actions as well as physiological signals of respiration and pulse.^81^ Another similar platform was developed with an Au coated PU-PDMS composite strain sensing nanomesh that could interestingly map strain on face while speech of different letters as shown in Fig. 4(b).^82^ Reduced graphene oxides (rGO) also have been utilized as the conductive layer to fabricate a three dimensional conductive stretchable electrode.^83^

Although metal-PU nanocomposite is a promising approach due to its high conductivity and stretchability, it often suffers from small fractures with the formation of visible cracks eventually causing shorting. In order to improve the mechanical properties, a reinforcing layer of poly(vinylidene fluoride) (PVDF) with a conductive layer of silver has been utilized as a stretchable electrode.^84–86^ A composite film of fluoroelastomer and silver flakes reinforced with electrospun PVDF nanofibers exhibited cyclic degradation (ΔR/R0) of only 0.56 after 5000 stretching cycles and stretchability upto 800%. This sensing electrode was attached to a skin tight suit to develop a multimodal physiological sensing platform for continuous biopotential monitoring during exercises without compromising signal quality [Fig. 4(c)].^85^ In another approach, graphene oxide (GO)-doped polyacrylonitrile (PAN) electrospun nanofiber yarns coated with conductive polypyrrole (PPY) was directly woven into textile to sense respiration, facile expressions, pulse, and a full range of human motion.^54,87^

### C. Wearable gas sensors for gauging body metabolism

High porosity and large surface to volume ratio allows electrospun nanofibers to absorb volatile material with ease and provide rapid response via color change or electrical signal. This capability has been explored to detect trace amounts of biomarkers in human breath and sweat samples. The obtained information can be processed and co-related to occurrence of specific diseases such as cancer, kidney disorder, and halitosis.^70,88^ A visual output-based sensing platform was fabricated by dual electrospinning of a composite polymeric nanofiber based on PAN co-functionalized with ionic liquids (ILs) and lead acetate (Pb(Ac)_2_) as effective H_2_S adsorbents and colorimetric dye, respectively. The yarn was sewn into patterned textile for fabrication of a colorimetric wearable fabric gas sensor that could reversibly detect NH_3_ gas molecules in the ppm-level. Moreover, this sensor could visually detect color change when exposed to a simulated exhaled breath of a halitosis patient, which typically contains about 1–1.8 ppm of H_2_S.^89^ In another work, a thermoplastic elastomer (TPE) polymer poly(styrene-block-butadiene-block-styrene) (SBS) was electrospun with silver nanoparticles (AgNPs) to produce a highly porous conductive elastic fiber with pH sensitive materials for sweat sensing. An artificial sweat sample was prepared by mixing NaCl (0.34 M), ammonium chloride (0.33 M), urea (0.08 M), acetic acid (0.04 M), and lactic acid (0.2 M) to achieve a pH value of 5.5 mimicking human sweat. As a proof of concept for wearable applications, a pair of these fibers was twisted to produce a rope shaped capacitor that could sense the artificial sweat sample both in relaxed and stretched states. Due to a highly permeable porous nanofibrous structure, this capacitor showed 17 times higher sensitivity for sweat sensing as compared to its nonporous counterpart.^90^ Alternatively, rGOs can also be used as building blocks. For example, rGO mixed nylon 6 electrospun nanofiber demonstrated sensitive response to NO_2_ (13.6%@1 ppm) at room temperature, capable of withstanding 5000 bending deformation resulting in the highly flexible and mechanically stable wearable sensor.^91^

Metal-organic frameworks (MOFs) are extensively used for chemical sensing owing to their abundant porosity, tunable chemical functionality, and high stability.^70,92^ A capacitive sensor for detection of H_2_S at room temperature was prepared by *in situ* integration of NO_2_-UiO-66 as a sensing material with electrospun polyacrylonitrile (PAN) nanofibers that were coated by a carbon nanotube (CNT) on both sides as an electrode. When tested with a mixture of various analytes (H_2_S, SO_2_, C_6_H_6_, CO, and NH_3_), significantly high selectivity for H_2_S was demonstrated due to the highly permeable nanofibrous structure. Moreover, remarkable sensitivity with detection limit reaching down to 10 ppb was observed with negligible reduction in sensitivity to detect even after five weeks. Moreover, the sensing performance was stable after different degrees of deformation, showing high potential to be integrated into electronic textiles as a gas mask.^93^

## IV. WEARABLE ENERGY STORAGE DEVICES

In Sec. III, we discussed biophysical and biochemical sensing applications of electrospun nanofibers. In this section, we will extend our discussion to essential building blocks in wearable platforms such as batteries, supercapacitors, rectennas, photovoltaics, and transistors, enabled from the use of electrospun nanofibers.

### A. Battery

The advancements in wearable electronics and devices are strongly correlated with the developments in the fields of semiconductors and microelectronics. Hence, wireless technologies, consumer electronics, communication systems, and the sensing platforms based on these developments have observed exponential growth in recent years. Despite of technological advancements, an increase in the demand for these devices has also triggered the need for miniaturization of these devices and improvements in their battery life. Since the invention of the first transistor in 1947 at Bell Labs, the dimensions of electronic circuits have decreased dramatically over the course of time, which paved the way for many sophisticated devices and products that are being used in our daily lives. Examples of such devices include personal computers, Internet of Things (IoT) devices, smart home appliances, and biomedical equipment. An enormous progress has been made to develop complex, yet small, and low-power electronics; however, battery life still remains one of the biggest design challenges in almost every aspect of wearable technology today.

**FIG. 5.**
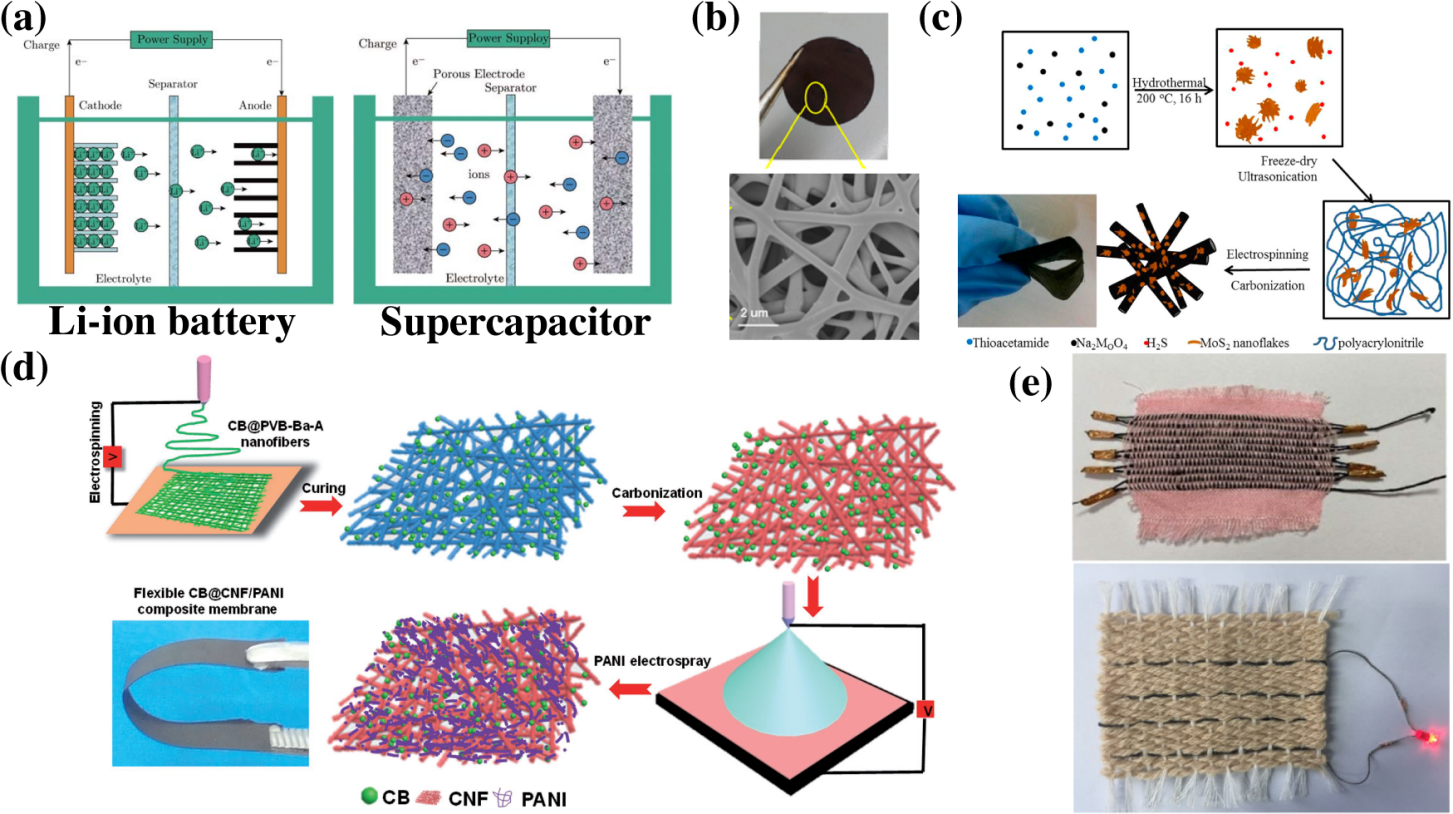
(a) Schematic diagram of the operating principle of a typical rechargeable Li-ion battery and a supercapacitor, (b) SEM image of a fused carbon fibrous mat based anode material for the Li-ion battery, (c) schematic illustration of the fabrication pathway of the MoS_2_/carbon composite, (d) schematic illustration of the electrospinning pathway of the PANI coated flexible supercapacitor, and (e) photos of fabricated nanofiber fabric supercapacitors. Reproduced with permission from Zhao *et al.*, Nano-Micro Lett. **3**, 62 (2011). Copyright 2011 Authors, licensed under a Creative Commons Attribution (CC BY) license. Copyright 2013 Authors, Wang *et al.*, ACS Appl. Mater. Interfaces **5**, 12275 (2013). Copyright 2013 American Chemical Society. Reproduced with permission from Zhao *et al.*, ACS Appl Mater. Interfaces, **6**, 4 (2014). Copyright 2014 American Chemical Society. Reproduced with permission from Iqbal *et al.*, Adv. Mater. Interfaces **4**, 1700855 (2017). Copyright 2017 Wiley-VCH GmbH. Reproduced with permission from Sun *et al.*, Materials **12**, 273 (2019). Copyright 2019 Authors, licensed under a Creative Commons Attribution (CC BY) license.^94,101,110,123,124^

Lithium–ion (Li–ion) batteries are predominantly used in wearable devices as they are safe to operate, lightweight, compact, demonstrate high charging capacity, and require low maintenance [Fig. 5(a)].^94,95^ Other common types of batteries found in wearable devices are alkaline, nickel–metal–hybrid (Ni–MH), and lithium–ion polymer (LiPo or Li–poly) batteries. It is plausible to say that for all types of batteries used in wearable devices, it is important to satisfy certain conditions for designing or selecting the optimal battery for a wide range of applications.^96^ Technical challenges that must be solved can be epitomized as the need for small, ultra-flexible, compact, highly efficient, long lifetime, and safe energy storage solutions. Rapid development of electrospinning methods has paved a way for the realization of electrospun nanofibers usage in soft electronics.^97,98^ Nanofibers have gained attention in recent years for making batteries due to various advantages, especially high porosity. Porosity is one of the most important engineering criteria for an efficient battery due to offering a high surface area that enhances the reaction rate and improves the capacity of both ion and electron storage. Porous nanofibers from electrospun nanoparticles and composites have shown improvements in lithium storage ability, charge–discharge kinetics, cyclic stability, and overall electrochemical performance.^99,100^ As a result, electrospun nanofibers are great candidates for making efficient, flexible, and stretchable batteries.

Wang *et al.* reported fused electrospun carbon fibrous mats used as high performance anode materials in Li–ion batteries to enhance specific capacity and electrochemical conductivity of carbon nanofibers, while simplifying the manufacturing process and further improving the battery charge capacity [Fig. 5(b)].^101^ To exemplify, obtaining an ultra-high battery capacity in the lithium-ion battery structure presented by Nan *et al.* has been attributed to attaining a large surface area, a well-developed porous structure, and a high nitrogen doping level.^102^ Additionally, other carbon-based electrospun nanofibers were also fabricated as anode materials^103,104^ and cathodes^105^ for lithium-ion batteries that can achieve very high rate capacities and stable electrochemical cycles. In another study, the authors have demonstrated a methodology to use nitrogen-based polymeric precursors in the fabrication of high-performance porous cathodes.^105^ Various other anode materials in lithium-ion batteries that have been synthesized and fabricated include ultrafine SnO_x_ particles,^106^ silicon core/C shell,^107^ silicon nanowires,^108^ and carbon nanofibers of high graphitization.^109^ Electrospun carbon nanofibers with embedded inorganic materials have been shown to provide electrochemical improvements in lithium-ion batteries. Among different inorganic additives, molebdenum disulfide (MoS_2_) is particularly a promising material for an anode due to its very high Li+ ion capacity. However, achieving uniform and efficient encapsulation of MoS_2_ in carbon nanofibers is a challenging task.^110,111^ In one approach, synthesized MoS_2_ nanoflakes were dissolved in PAN to electrospin hybrid PAN/MoS_2_ nanofibers. Next, the hybrid nanofiber assembly was carbonized to obtain a MoS_2_/carbon composite [Fig. 5(c)].^110^ PVP was also used as another precursor for carbonization to fabricate the MoS_2_ nanoplate embedded carbon nanofiber.^111^ Electrospinning facilitates embedding nanoflakes/nanoplates of functional materials into the structure of conductive nanofibers to fabricate battery materials with unique structure. These composite structures not only offer modulation of rate performance but also demonstrate extreme confinement of reaction into these flexible, ultrathin nanostructures for improved battery performance.

### B. Supercapacitor

Conventional batteries rely on the electrochemical reactions to convert chemical energy into electrical energy. Despite the advantage of large capacitance, conventional batteries suffer from problems such as slow charge-recharge rate, low energy density, low power density, and short recharge lifetime.^112^ They are also bulky, heavy, and rigid, whereas soft and flexible electrodes are essential for developing flexible supercapacitors. On the other hand, capacitors have a very high charge rate; however, low capacitance limits their use as an energy storage device. Supercapacitors have emerged as a promising candidate for energy storage devices, owing to their fast charge–discharge rate, high power density, long cycling life, and wide working temperature range.^113^ Supercapacitors are categorized mostly into two types, electrical double-layer capacitors (EDLCs) and pseudocapacitors.^114,115^ To achieve high electrical capacitance, EDLCs use a nanometer scale self-formed (self-assembled) electrolytic double layer that forms between the electrode and the electrolyte as their primary source of capacitance. Increasing the surface area of the electrode will increase the capacitance. On the contrary, the pseudocapacitors exploit Faradaic reactions at the interface between the electrolyte and electrodes to achieve large capacitance.^116^ Since capacitance is proportional to the surface area of the electrode, a highly porous nanostructured electrode or interlayer can greatly enhance the charge density and capacitance of the supercapacitor. Moreover, using nanostructures will also achieve a large area to volume ratio, making the supercapacitors lightweight. Nanocarbon and conductive polymers are most extensively used flexible materials for designing a supercapacitor electrode.^117^ Electrodes made with nanocarbon-based materials exhibit high energy storage capacity, power density, and longer lifetime, whereas polymer-based electrodes show reduced energy capacity, but superior flexibility.^118,119^

Electrospinning, therefore, has emerged as a suitable technique for fabrication of flexible supercapacitor electrodes due to its electrospun nanofibers' high surface-volume ratio, intrinsic flexibility, and elasticity, as well as the easily controllable fiber properties by tuning the electrospinning parameters. Electrospinning is a modular technique that provides the ability to tune fiber properties by controlling processing parameters, as well as a switch core material as required for appropriate application. Polyaniline (PANI), being the most commonly used material for the flexible supercapacitor, can be electrospun to form a nanofiber network as an electrode.^120,121^ The fabricated flexible supercapacitor exhibits a specific capacitance of 134 F/g, a power density of 0.8 W/g at 0.8 V, and an energy density of 11.91 mWh/g. It maintains 85.6% of its capacity after 20 000 charging cycle and negligible degradation after 500 bending cycles. The supercapacitor made with electrospun vanadium/cobalt oxides (VCO) and carbon nanofiber electrodes^122^ has a specific capacitance of 1.83 F/cm^2^, a current density of 8 mA/cm^2^, and an energy density of only 44.2 *μ*Wh/cm^2^. To combine the advantage of multiple materials, hybrid electrodes are also generated with electrospinning. Researchers have used techniques such as electrospray and chemical polymerization to form a PANI coating over electrospun carbon nanofibers. The hybrid structure has the advantage of the high surface area and better electrical conductivity as well as structural stability and tolerance to external deformation.^116,123^ The carbon nanofiber coated with electrosprayed PANI has a specific capacitance of 501.6 F/g and a current density of 0.5 A/g [Fig. 5(d)]. The capacity remains 915 after 5000 charging cycles and shows no significant decrease to bending. The carbon nanofiber coated with chemical polymerization PANI has a specific capacitance of 234 F/g, an energy density of 32 mWh/g, and a power density of 0.5 W/g.

In another approach, the core spun yarn electrode was fabricated by electrospinning and woven into a fabric to make a wearable supercapacitor [Fig. 5(e)]. The PAN/GO composite electrospun nanofiber was wrapped around a nickel (Ni) coated commercial cotton yarn (NCY). Ni coating provides high tensile strength and good conductivity. The resulting fabricated yarn supercapacitor was woven as an energy storage fabric.^124,125^ The woven supercapacitors were highly flexible and elastic due to their fabric property, demonstrating the specific capacitance of 28.34 mF/cm^2^ and 25.31 F/g. The nanofibers grown on NCY provide more active sites to improve the transportation rate of electrons. The core NCY was responsible for excellent capacitance retention even after 1000 cyclic voltammetry cycles. This work presents a unique strategy to fabricate the weavable nanofiber based supercapacitor toward powering of a wide range of wearable devices.

## V. WEARABLE ENERGY HARVESTING DEVICES

### A. Photovoltaics

Photovoltaic devices can absorb photons and generate electrical charge carriers with the absorbed energy. Generated electrons and holes then move toward cathodes and anodes, respectively, creating an electric current. With the rapid expansion of IoTs, flexible solar cells have become a promising candidate for wearable power source. Electrospun nanofibers are suitable as wearable power sources, attributing to their low-cost, ease of fabrication, and tunable physicochemical characteristics.

**FIG. 6.**
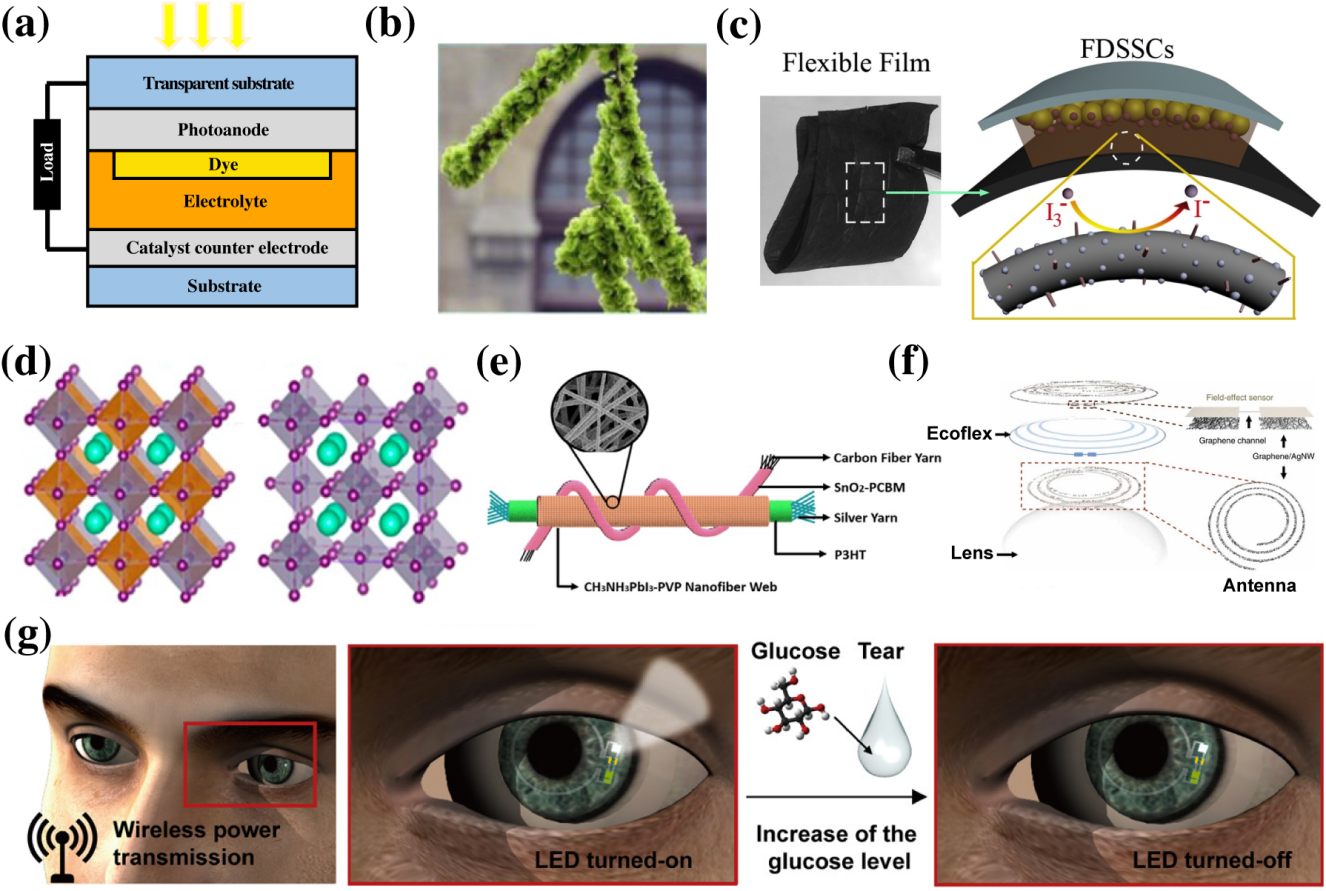
(a) Schematic structure of DSSCs, (b) flower-like DSSC with the nanofiber counter electrode, (c) flexible DSSC yarn and film with the nanofiber counter electrode, (d) single and double perovskite atomic structure, (e) perovskite yarn solar cell using CH_3_NH_3_PbI_3_–PVP nanofiber as an active layer, (f) schematic illustration of a stretchable transparent antenna, and (g) schematic illustration of a soft, smart contact lens. Reproduced with permission from Nien *et al.*, Vacuum **167**, 47 (2019). Copyright 2019 Elsevier. Reproduced with permission from Li *et al.*, Electrochim. Acta **280**, 94 (2018). Copyright 2018 Elsevier. Reproduced with permission from Song *et al.*, Mater. Res. Bull. **118**, 110522 (2019). Copyright 2019 Elsevier. Reproduced with permission from El-Shazly *et al.*, ACS Appl. Nano Mater. **2**, 7085 (2019). Copyright 2019 American Chemical Society. Reproduced with permission from Li *et al.*, Sol. RRL **4**, 2000269 (2020). Copyright 2020 Wiley-VCH GmbH. Reproduced with permission from Kim *et al.*, Nat. Commun. **8**, ▪ (2017). Copyright 2017 Authors, licensed under a Creative Commons Attribution 4.0 International license (CC BY 4.0). Reproduced with permission from Park *et al.*, Sci. Adv. **4**, eaap9841 (2018). Copyright 2018 Authors, licensed under a Creative Commons Attribution (CC BY) license.^127,138,140,141,146,161,162^

Multiple examples of dye-sensitized solar cell (DSSC) incorporating an electrospun nanofiber as a component have demonstrated increased efficiency in wearable applications. A typical DSSC consists of a transparent conductive substrate, a photoanode, the dye, an electrolyte, and a counter electrode (cathode) [Fig. 6(a)].^126,127^ The dye and electrolyte are critical components, where the optoelectronic process occurs. Unlike other solar cells, where the optical process happens through photo-induced charged generation in the PN junction of the solar cell during light absorption, the mechanism is more complex in DSSC. The dye particles of a DSSC lose their electrons upon light excitation, consequently becoming oxidized. The generated electrons travel to the counter electrode via an external circuit whereby it reduces the electrolyte. The electrolyte then passes the electrons back to the dye and finishes the current loop. Traditional high power conversion efficiency (PCE) DSSCs have liquid electrolyte and often suffer from electrolyte leakage, evaporation, or electrode corrosion, which significantly reduce the lifetime of a solar cell.^128^ In a study, a layer of electrospun nanofibers was used to absorb the electrolyte to address the afore-mentioned problems.^127,129–132^ High porosity and surface to volume ratio enable the nanofiber to hold large quantities of the electrolyte, significantly reducing the rate of electrolyte leakage or evaporation. Moreover, DSSCs with nanofiber components retain their original PCEs even after a month, whereas the standard DSSCs with liquid electrolyte showed high reduction in their efficiency.^129–131,133^ The electrospun nanofiber offers an inexpensive and efficient alternative to other electrolyte techniques such as a gel electrolyte or a noble metal particle doped electrolyte. The inter-connected structure of the nanofiber allows them to maintain charge carrier mobility, rendering them semiconductor-like property, which helps in transportation of electrons and holes to the electrodes before they can recombine.^134^ With optimal composition and the fabrication process, the nanofiber electrolyte demonstrates similar, and in some cases better PCE than the liquid electrolyte doped with metal particles.^127,135^ Moreover, the porous nanofiber creates an excellent scattering architecture that prolongs the effective travel length of light within the active layer of DSSC,^136^ where more light absorption results in better efficiency.

The counter electrode is another component of DSSC that can be made with electrospun nanofibers. The counter electrode collects holes generated in the active layer, receives electrons from the external circuit, and catalyzes the redox reaction in the active layer. A common approach to generate flexible counter electrodes uses deposition of platinum (Pt) on transparent conductive materials for both carrier mobility and electrocatalytic capacity.^137^ Pt is extremely expensive and rare and, thus, limits their large-scale application. On the other hand, electrospun nanofibers are cheap and easy to fabricate. Moreover, efficient carrier mobility of nanofibers enables them to transport charge carriers between the active layer and the external circuit. Additionally, the large surface area of the nanofiber can compensate for its inherent low electrocatalytic activity.^138^ In another study, MoS_2_ and Bi_2_S_3_ were deposited on carbon nanofibers to form the counter electrode for DSSC using the hydrothermal method. These DSSCs demonstrate the PCE of 8.46%^138^ [Fig. 6(b)] and 7.64%,^139^ respectively, both exceeding the efficiency of the Pt counter cathode DSSC. Alternatively, MoS_2_ coated carbon nanofibers can be fabricated by directly bonding to a fabric to achieve a superflexible photovoltaic device with an efficiency of 5.08% [Fig. 6(c)].^140^

Perovskite photovoltaics is another kind of solar cell that is promising for wearable applications [Fig. 6(d)]. A typical perovskite solar cell has the advantage of low electron–hole binding energy, high charge carrier mobility, and long carrier lifetime. These advantages provide perovskite solar cells with high charge carrier generation rate and low recombination rate, thus drastically increasing their PCE compared to other types of solar cells.^141^ Perovskite solar cells made with organic–inorganic hybrid materials (MBX_3_) can reach efficiency as high as 23%.^142^ However, flexible perovskite solar cells exhibit low efficiency, shorter lifetime, and loss of potency after bending and twisting.^143^ Nanofibers can enhance the light harvesting process by minimizing the incident light reflection and maximizing the internal reflection of light inside the active layer. In a study, CH_3_NH_3_PbI_3_–PVP fibers fabricated with electrospinning were used as the active layer [Fig. 6(e)].^141^ With optimized electrospinning voltage, humidity, and time, fabricated flexible perovskite solar cells achieved a PCE of 15.7%. This wearable solar cell retained 99.5% of its original PCE after 750 bending cycles and 90% of the original value after 200 h. Similarly, CsPbX_3_ (X = Cl, Br, and I) nanofibers were also studied for organic-free perovskite solar cells, reporting a highest efficiency of 7.79%.^144^ Another critical challenge for wearable perovskite solar cells arises due to the toxicity from the use of lead, severely limiting their applications. Tin (Sn) has been used as a substitute; however, commonly used materials, such as CsSnX_3_ and Cs_2_SnX_6_, lack air instability and possess large bandgap.^145,146^ Electrospun Cs_2_SnX_6_ nanofibers show minimal degradation under air condition, and their bandgap can be tuned to an ideal value of 1.5 eV.^146^

### B. Rectenna

Energy harvesting from ambient radio waves is another way to generate energy to power the wearable devices. The rectenna, introduced by Raytheon Co. in 1963,^147^ is one such device, and it refers to the combination of an antenna, which receives radio waves, and a rectifier, which converts RF power into DC signals. They are commonly called radio frequency energy harvesting (RFEH) systems. Since their introduction in the late 1950s,^148^ RFEH systems have been extensively developed.^149–151^ Their main objective is to obtain the maximum PCE, determined by the ratio of the maximum output power of the device to the incident input power, where the input power is the sum over all or a range of wavelengths and commonly fixed at 100 mW/cm^2^. A rectenna typically consists of an antenna with a matching circuit and a diode rectifier.

Consequently, the PCE performance of a rectenna will be mainly influenced by the desired frequency range, bandwidth, the matching circuit, and the diode topology. The antenna dimensions are usually of the same length scale as the targeted frequency. Therefore, for microwave frequencies, the antenna is relatively large (cm to mm scale), yet not a problem for wearable or textile-based applications, because of the large available surface area. The matching circuit will determine the energy transfer efficiency from the antenna to the rectifier diode. This circuit is based on several discrete inductors and capacitors in series and/or parallel to match the input impedance seen from the antenna to the impedance of the rectifier and load. The rectifier is mainly based on diodes and capacitors with different topologies like in series, shunt, and single stage voltage doubler, among others. Different topologies will generally provide different PCEs, voltage output levels, design complexities, and sizes.

The use of lightweight, flexible, and conformable materials has been reported in the field of RFEH systems.^152–160^ They have reported up to 70% of PCE for a different range of wavelengths. Yet, these rectennas are still bulky and thick (>1–3 mm), offering limited stretchability while trying to achieve a good RF performance. Large antenna size often sacrifices breathability of underlying skin. The humidity and temperature dependence also influences antenna performance due to change in material dielectric properties. Another challenge is ensuring effective electrical connection between the flexible antenna and the hard matching components.

Electrospun nanofibers based RFEH systems have been reported to demonstrate excellent mechanical and electrical properties to provide stretchability, transparency, and conformability to the antenna and the rectenna's interconnects. Porosity of the antenna allows for breathability, which is a key for any wearable and implantable applications. In a study, a soft, smart contact lens was reported composed of a hybrid substrate, functional devices (rectifier, LED, and glucose sensor), and transparent, stretchable conductive nanostructures (for antennas and interconnects) [Figs. 6(f) and 6(g)].^161,162^ The system showed superior mechanical durability on curvilinear surfaces such as contact lens, where the system was successfully stretched up to 30% in tensile strain. A suspension of Ag nanoparticle ink in ethylene glycol was electrospun to form a network of continuous Ag nanofibers (a maximum thickness of 2 *μ*m) patterned with a single loop structure, obtaining overall antenna dimensions of 12 mm in diameter and 0.5 mm in width. The rectenna achieved a PCE of 21.5% at 50 MHz, transmitted at 5 mm. The constructed Ag nanofibers had an average sheet resistance of 0.3 Ω per square and a transparency of 71% at 550 nm, which are good values compared to previously reported in the literature.^148^ In another study, a highly stretchable and transparent wireless electronic system composed of Ag nanofibers coil antennas and components for power transfer and information communication was reported.^163^ High precision patterned Ag NFs electrodes served as porous conductors with nanowires forming percolation networks. PVA nanofibers were first made using electrospinning followed by magnetron sputtering of silver. Lithography was used to pattern them into coils and other shapes. They performed in-depth studies on stretchability vs PCE levels, by modifying the fabrication technique (using different Ag NF densities, electrospinning durations, and orientations of the NFs), frequency, input impedance, and the number of turns in the coil antenna, among others. They achieved a good PCE level up to 50% at 10 MHz with a strain of 100% and transmitted up to 2 cm away. Approaches other than electrospinning have been used to make antennas using Ag nanofibers that can be scaled easily using electrospinning.^164,165^

### C. Nanogenerators

Another source of generating or harvesting power is from mechanical motion. Peizoelectric materials are well known to convert mechanical deformation into charge generation. Newer approaches use triboelectricity, where the contact between two dissimilar material results in electrification of these materials in a term defined triboelectric effect. For providing a more sustainable, small-scale, and high efficiency energy source, nanogenerators based on nanomaterials have been developed. Nanogenerators (NGs) are compact and lightweight devices that convert mechanical thermal or other alternative energy source into electricity. Nanogenerators based on piezoelectricity and triboelectricity have been developed for mechanical energy harvesting while pyroelectricity and thermoelectric effects have been investigated in order to harvest thermal energy.^166^ The conversion from thermal or mechanical energy into electrical energy creates an opportunity for the nanogenerators to be used in self-powered sensors and systems for wearables. The electrospinning technique has been largely utilized to construct fiber-structured nanogenerators. Recently, using a one-pot electrospinning method, an all-fiber triboelectric nanogenerator (TENG) has been reported.^167^ The operation principle of TENGs is based on what is called contact electrification.^168^ It is, therefore, possible to generate electrical energy continuously using TENG devices, which are based on the triboelectric friction effect. Being a cost-effective and versatile technique, electrospinning can be utilized to process micro- and nanofiber structures, which then turn into materials with high surface area and porosity. These features make electrospun nanofiber based TENGs ideal candidates as energy harvesters. Ghosh *et al.* reported 1D polymer nanofibers for wearable nano-tactile sensor applications.^169^ As the performance of the energy conversion using electrospun based materials mostly depends on the material structure and design, triboelectric nanogenerators based on PVDF nanofibers have also been investigated. Taking surface morphology of the PVDF nanofibers into account, churros-like PVDF nanofibers were fabricated in order to control the solvent evaporation kinetics. Coupled with the modulation of the changes in the relative humidity, enlargement of the surface area of the PVDF nanofibers helps enhance the performance of the TENG devices such that the output voltage and power density could go up as high as 234 V and 1738 *μ*W/cm^2^, respectively. Khalifa *et al.* reported a study on electrospun PVDF nanofibers with embedded polyaniline (PANI)/graphitic carbon nitride nanosheets (g-C_3_N_4_) blend nanocomposite fibers (PPBF)-based piezoelectric nanogenerators.^170^ It has been shown that the PPBF nanogenerator is much superior in performance compared to the PVDF nanofibers. The improvement in the voltage output obtained from the nanogenerators is attributed to the *β*-phase content. The piezoelectric potential inside the fibers was tested by repeatedly tapping on the nanogenerator, which then results in creation of piezoelectric potential. The electrospinning technique is shown to boost the piezoelectricity of the PVDF material. Similarly, You *et al.* presented a piezoelectric nanogenerator (PENG) from aligned poly(vinylidene fluoride-*co*-trifluoroethylene), denoted as P(VDF-TrFE) nanofibers.^171^ This PENG can achieve a high output voltage of 12 V and a short circuit current of 0.15 *μ*A with capability to light up six LEDs and harvest the energy from human walking. Recently, newer approaches using biodegradable and bioresorable materials, such as poly-l-lactic acid (PLLA), have also been used to generate power.^172^ Electrospinning improved the piezoelectric coefficient of these materials. These nanogenerators demonstrate great potential of self-powered devices and wearable energy harvesters suitable for wearable electronic equipment.

## VI. TRANSISTORS

**FIG. 7.**
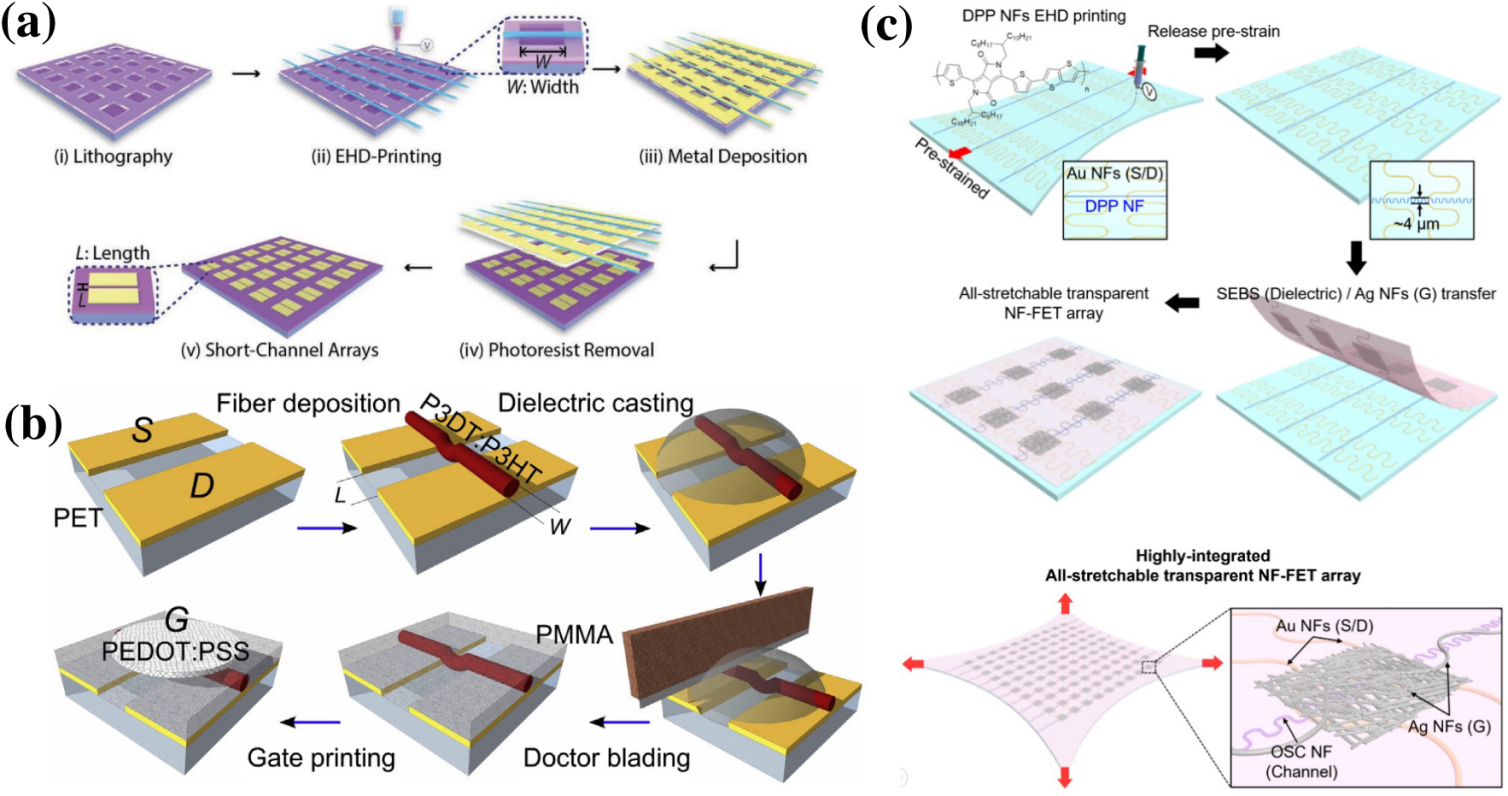
(a) Schematic diagram of suspended nanofiber lithography, (b) single nanofiber as the FET channel, and (c) stretchable nanofiber transistor. Reproduced with permission from Liu *et al.*, Adv. Funct. Mater. 2109254 (2021). Copyright 2021 Wiley-VCH GmbH. Reproduced with permission from Manuelli *et al.*, Org. Electron. **15**, 1056 (2014). Copyright 2014 Elsevier. Reproduced with permission from Kim *et al.*, Nano Lett. **21**, 5819 (2021). Copyright 2021 American Chemical Society.^181–183^

Field effect transistors (FETs) are the key building blocks of any integrated circuits. The FET consists of three terminals: source, drain, and the gate, where the gate terminal acts like a control switch that controls the formation of an active conductive channel between the drain and source.^173^ When an electric field is applied onto the gate, the channel between the drain and source widens or narrows accordingly, thus adjusting the density of charge carriers between the drain and source.^174–176^ In a digital view of the transistor, it behaves like a switch, where the gate controls the On and Off states. The key metrics are the On/Off current ratio and the speed of switching. In the analog view of the transistor, it behaves more like a transconductor, where the gate controls the current level between the drain and source. Conventional FETs are made with solid-state semiconductor channels lacking required flexibility to be applied for wearable FETs and are generally fabricated using expensive photolithographic processes. There have been significant progress in the fabrication of intrinsically stretchable FETs by employing thermoplastic elastomers in combination with organic semiconductors such as PEDOT:PSS and others.^177,178^ However, such FETs suffer poor mobility, lack of breathability, and also some stretching related artifacts. In this context, electrospinning has high potential to be an alternative strategy to make the transistor. Careful choice of the flow rate and polymer viscosity allow preferential orientation of polymer chains that has been reported to enhance the charge carrier mobility over three orders of magnitude.^179^ Electrospun nanofibers can be precisely positioned onto the substrate and serve as a sacrificial mask to create semiconducting channels in FETs, substituting the photoresist and lithography process.^180^ The nanofiber blocks deposited gold and create a gap for the channel when removed. The length of the channel was determined by the thickness of the nanofiber and ranged from 350 to 1000 nm. With the suspended nanofiber lithography technique, the channel length as short as 48 nm could be achieved [Fig. 7(a)].^181^

Other than masks for material deposition, electrospun nanofibers can be used as the building material for flexible transistors. Utilizing the near-field electrospinning method, a single nanofiber was precisely placed across a drain and source electrode to form a single-wire nanofiber channel [Fig. 7(b)].^182^ Such fabricated flexible FETs reached a stable state after 100 bending cycles and displayed a bending tolerance up to 1000 cycles. Similarly, all components of the transistor, including the drain and source electrodes, the channel, and the gate electrode, were made with electrospun nanofibers to achieve total flexibility and stretchability^183^ as shown in Fig. 7(c). The gold drain and source electrodes, polymer channel, and the silver gate electrode were fabricated by either near-field electrospinning or mass electrospinning.

## VII. SUMMARY AND OUTLOOK

Since the emergence of electrospinning, remarkable progress has been made in utilizing this technique for multiple applications especially in the realm of wearable devices electronics. First of all, there is a rich diverse set of materials that are amenable for electrospinning including materials that are biocompatible, biodegradable, inert, dielectric, piezoelectric, and conductive. This review illustrated recent examples of electrospun nanofibers that are integrated with human skin and/or clothing to develop smart wearable devices (Table I). We have classified wearable devices into two broad categories on the basis of their applications in the field of physiological sensors and energy devices. Electrospun materials offer several advantages over conventional bulk materials. For instance, the high surface to volume ratio endows them with high porosity and breathability, whereas biocompatibility can be achieved by using appropriate blend of polymers. Specifically, conductive electrospun nanofibers provide high surface area electrodes that not only provide flexibility but also performance improvements such as rapid charging and high storage capacity in the case of energy storage and harvesting devices. Moreover, electrospinning provides an inexpensive and more user-friendly alternative to photolithography for realizing nanoscale transistor morphologies with superior electronic transport. Clearly, electrospinning is a promising strategy that will further establish its claim as a versatile, feasible, and inexpensive technique to fabricate wearable devices in the forthcoming years.

**TABLE I.** Use of electrospun nanofibers for wearable devices.

Device	Electrospun polymer	Functional materials	Operating mechanism	Application in wearables	References
Electro-physiological signal sensor	PVA, PU, annealed PR, PAN, TPU, PVDF/PEDOT/PSS	Au, AgNWs, CB, rGO	Epidermal electrodes based bio-potential sensor	ECG, EMG recording	67–76
Motion sensors	PU, PVDF, PPY, PAN	CNT, rGO, silver flakes	Electrical resistance based strain senor		78–87
Gas sensors	PAN, SBS, nylon 6,	ILs, Pb(Ac)_2_, AgNPs, rGO, NO_2_-UiO-66, CNT	Colorimetric gas sensor	Output based sensing platform to process the response and corelate to occurrence of specific diseases such as cancer, kidney disorder and halitosis	89–93
Batteries	PAN, PEO, PVP	MoS_2_, SnO_x_,	Porous structure enhances reaction rate.	Efficient, flexible and stretchable batteries	101–110
Supercapacitor	PAN, PANI, VCO, carbon nanofiber	GO, Ni	Nanostructured interlayers to enhance charge density and capacitance	Flexible and weavable nanofiber based supercapacitor	120–125
Dye-sensitized solar cell	PVP, carbon nanofiber,	Pt, MoS_2_, Bi_2_S_3_, CH_3_NH_3_PbI_3_	Porous nanofiber based scattering architecture to help electron transportation	Flexible solar cells for wearable power source	127 and 129–135
Perovskite photovoltaics	PVP, CsPbX_3_, Cs_2_SnX_6_ (X = Cl, Br, and I)	CH_3_NH_3_PbI_3,_	Flexible nanofiber based solar cells	Flexible perovskite solar cells with higher efficiency and longer lifetime	143–146
Rectenna	AgNFs, PVA	PDMS, Cu	Porous nanofiber based structure for breathable and flexible wearable rectennas.	Flexible, stretchable antenna, soft smart contact lens	161–165
Nanogenerator	PVDF, PANI	g-C_3_N_4_, trifluoro-ethylene	Contact electrification based piezo- and tribo-electric nanogenerators.	Self-powered devices and wearable energy harvesters	166–172
Transistor	Au, Ag, P3DT:P3HT, DPP	SEBS	Nanofibers can be used as building materials for channel, drain, source and gate electrodes to achieve lithography free fabrication.	Mask, drain/source/gate electrode	173–183

Looking ahead, broadening of the material choice and improving the ease of integration with human physiology are critical aspects for advancement of electrospun-based devices. For example, innovative materials that can improve the durability of the electrospun tattoos or skin patches, making them more biocompatible, while providing multifunctional capabilities such as sensing and actuation, while also providing high breathability and reduce inflammation or irritation in the skin will be sought. New material options for flexible polymers and electrode materials that can be electrospun to improve the performance of battery and Supercapacitors are needed. With regard to transistors, electrospinning is still at early stage. Most devices fabricated using electrospinning cannot be fabricated en masse and are not scalable for universal applicability. We expect electrospun transistors to complement the existing silicon based semiconductor electronics industry specifically for wearable and biomedical applications. Applications of electrospun nanofibers are probably for wearable applications, where large area transistors are acceptable and also breathability and flexibility are more important than the individual transistor performance. New strategies offering seamless integration of wearable devices with power source and communication components are key to improve overall functioning of any wearable system and will need further investigation especially when it comes to electrospun devices.

Another overlooked aspect for wearable devices is their esthetic appearance. Strategies should be developed to make more compact wearable devices with reduced overall thickness. In addition, transparent materials should be utilized with an overall goal to develop an almost invisible wearable device. Esthetically pleasing wearable device can be easily translated into fashionable accessories and addresses multiple challenges in the field of personalized healthcare and energy. We believe electrospun porous devices offer such an opportunity; however, there is still room for innovation in this regard.

## Data Availability

Data sharing is not applicable to this article as no new data were created or analyzed in this study.
